# Migratory phase of *Litomosoides sigmodontis* filarial infective larvae is associated with pathology and transient increase of S100A9 expressing neutrophils in the lung

**DOI:** 10.1371/journal.pntd.0005596

**Published:** 2017-05-09

**Authors:** Gregory Karadjian, Frédéric Fercoq, Nicolas Pionnier, Nathaly Vallarino-Lhermitte, Emilie Lefoulon, Adélaïde Nieguitsila, Sabine Specht, Leo M. Carlin, Coralie Martin

**Affiliations:** 1 Unité Molécules de Communication et Adaptation des Microorganismes (MCAM, UMR 7245), Sorbonne Universités, Muséum national d’Histoire naturelle, CNRS, Paris, France; 2 Institute for Medical Microbiology, Immunology & Parasitology (IMMIP), University Hospital of Bonn, Bonn, Germany; 3 Inflammation, Repair and Development, National Heart & Lung Institute, Imperial College London, London, United Kingdom; University of Würzburg, GERMANY

## Abstract

Filarial infections are tropical diseases caused by nematodes of the Onchocercidae family such as *Mansonella perstans*. The infective larvae (L3) are transmitted into the skin of vertebrate hosts by blood-feeding vectors. Many filarial species settle in the serous cavities including *M*. *perstans* in humans and *L*. *sigmodontis*, a well-established model of filariasis in mice. *L*. *sigmodontis* L3 migrate to the pleural cavity where they moult into L4 around day 9 and into male and female adult worms around day 30. Little is known of the early phase of the parasite life cycle, after the L3 is inoculated in the dermis by the vector and enters the afferent lymphatic vessels and before the moulting processes in the pleural cavity. Here we reveal a pulmonary phase associated with lung damage characterized by haemorrhages and granulomas suggesting L3 reach the lung via pulmonary capillaries and damage the endothelium and parenchyma by crossing them to enter the pleural cavity. This study also provides evidence for a transient inflammation in the lung characterized by a very early recruitment of neutrophils associated with high expression levels of S100A8 and S100A9 proteins.

## Author summary

*Mansonella perstans* is a widespread human filarial parasite in Africa responsible for pleural and peritoneal cavity filariasis. Compared to other filarial parasites such as *Wuchereria bancrofti*, *Brugia malayi*, and *Loa loa*, the biology of *M*. *perstans* is poorly known. The blood-feeding vectors inject infective larvae (L3) into the host skin during a blood meal. Depending on the species, the L3 will then migrate to its specific site. In the murine model of filariasis *Litomosoides sigmodontis* L3 also reach the pleural cavity where they moult twice then mate and produce microfilariae. Migration patterns from the skin to the pleural cavity are partially known and involve a lymphatic phase. Here we present a sequential analysis of L3 infection from their inoculation to day 8 when they are settled in the pleural cavity, revealing the presence of L3 in the lung. Pulmonary damage including haemorrhages and granulomas is also observed suggesting that L3 could migrate to the pulmonary circulation and capillaries from where they could exit the lung to reach the pleural cavity. This induces a local inflammatory response characterized by neutrophil activation and upregulation.

## Introduction

Blood-feeding vectors inject filarial infective larvae (L3) into the host skin during a blood meal. Most of the filarial species migrate through the host’s body from the skin to their definitive niche, mainly the lymphatic system, the serous cavities, the cardiopulmonary system, or connective tissues [1]. Rodent models are helpful to investigate the migratory routes of L3 showing an early pulmonary phase for the human *Brugia malayi*, and for the main animal models of filariasis *e*.*g*. *Brugia pahangi*, *Acanthocheilonema viteae* and *Litomosoides sigmodontis* [2]. The latter is the sole filaria to undergo full development in immunocompetent BALB/c mice [3]. In addition *L*. *sigmodontis* share various biological features with the human *Mansonella perstans* such as a moulting process into stage 4 within 9–10 days, adults living in the serous cavity including the pleural cavity, blood circulating microfilariae [3–5]. Both are considered as ‘derived filariae’ [6].

*M*. *perstans* is a vector-borne human filarial nematode, transmitted by *Culicoides* (biting midges) [7]. It is responsible for serous cavity filariasis in humans, including pleural cavity infection [8]. However, very little is known about the biology of this parasite and migration patterns from skin to serous cavities remain unidentified. The incidence of mansonellosis (due to *M*. *perstans*) is underestimated as it is considered of less pathogenic importance when compared to onchocerciasis, lymphatic filariasis and loasis. The pathogenicity of *M*. *perstans* infection has been recently reconsidered in various studies as it is estimated that almost 120 million people are infected by *M*. *perstans* in Africa [8–10]. Although infections with this parasite often remain asymptomatic, a vast range of symptoms can also be provoked, e.g. subcutaneous swellings, aches, pains, skin rashes, hormonal disturbances and hypereosinophilia [8].

In 1967, Wenk [11] hypothesized that the infective larvae (L3) may pass through the lung to reach the pleural cavity for both *Litomosoides carinii* (since synonymised as *L*. *sigmodontis*) and *Acanthocheilonema perstans* (since synonymised as *M*. *perstans*). Indeed, the occurrence in the lung has been established for many filarial nematodes (Onchocercidae [6]) species in a large range of hosts including birds, crocodiles, dogs, and humans (Table 1). However, most of these studies documented the presence of either adult worms or microfilariae but very rarely L3. *L*. *sigmodontis* L3 were observed in the mechanically disrupted lungs of BALB/c mice at the second day post inoculation [12] and earlier in their natural hosts the cotton rats [11]. Furthermore, the recent observation of granulomas, mainly constituted of T cells and macrophages, in the lung of *L*. *sigmodontis* infected mice at day 8 post inoculation also reinforce this hypothesis [13]. However, the presence of an inflammatory reaction in the pulmonary tissue has not yet been addressed. We aimed to shed light on the biology of *L*. *sigmodontis* infective larvae in this early pulmonary phase of infection. Our results support the arrival of the larvae in the cardiopulmonary system before entering the pulmonary tissue and reaching the pleural cavity. They also reveal a transient inflammation characterized by a fast recruitment of neutrophils into the lung associated with high expression levels of S100A8 and S100A9 proteins. S100A9 (also known as calgranulin B) complexes with S100A8 to form a heterodimer called calprotectin. Both are small calcium-binding proteins that are highly expressed in neutrophil cytosol and are found at high levels in the extracellular medium during inflammatory conditions. In particular, S100A9 is expressed in non-small cell lung cancer [14].

**Table 1:** Filarial nematodes with evidences of a pulmonary location.

Subfamilly	Parasite	Host (experimental/accidental)	Described stage	Pulmonary location	Ref
**Oswaldofilariinae**	*Oswaldofilaria bacillaris* (Molin, 1858)	Crocodylidae	Adults	Whole lungs	[15]
	*Oswaldofilaria chlamydosauri* (Breinl, 1913)	Agamidae, (Agamidae)	Adults	Whole lungs	[16]
	*Conispiculum flavescens* (Castellani et Willey, 1905)	Agamidae	Adults	Whole lungs	[17]
	*Piratuba queenslandensis* Mackerras, 1962	Varanidae	Adults	Whole lungs	[16]
	*Piratuba varanicola* Mackerras, 1962	Varanidae	Adults	Whole lungs	[16]
**Dirofilariinae**	*Dirofilaria immitis* (Leidy, 1856)	Canidae	Adults, mf	Pulmonary arteries, bronchus	[18]
	*Dirofilaria immitis* (Leidy, 1856)	(Humans)	Adults	Whole lungs	[19]
	*Dirofilaria spectans* Freitas et Lent, 1949	Mustelidae	Adults	Pulmonary arteries	[20]
	*Foleyella dolichoptera* Wehr et Causey, 1939	Ranidae	Adults	Lung tissue	[21]
**Onchocercinae**	*Acanthocheilonema spirocauda* (Leidy, 1858)	Phocidae	Adults	Pulmonary arteries, Whole lungs	[22]
	*Acanthocheilonema viteae* (Krepkogorskaya, 1933)	nd, (Rodents)	L3, L4	Whole lungs	[2]
	*Brugia pahangi* (Buckley & Edeson, 1956)	nd, (Rodents)	L3, L4, Adults	Whole lungs, pulmonary arteries	[2,23,24]
	*Brugia malayi* (Brug, 1927)	Humans	mf	Whole lungs	[25]
	*Brugia malayi* (Brug, 1927)	(Rodents)	L4, Adults, mf	Whole lungs, pulmonary arteries	[2,24]
	*Brugia buckleyi* Dissanaike et Paramananthan, 1961	Leporidae	Adults	Pulmonary arteries	[26]
	*Cardiofilaria chabaudi* Dissanaike et Fernando, 1965	Cuculidae	Adults	Whole lungs	[27]
	*Deraiophoronema evansi* (Lewis, 1882)	Camelidae	Adults	Pulmonary arteries	[28]
	*Monanema globulosa* (Muller ans Nelson, 1975)	nd, (Rodents)	L4, Adults	Whole lungs, pulmonary arteries	[2]
	*Monanema martini* Bain, Bartlett and Petit, 1986	nd, (Rodents)	L3, L4, Adults	Pulmonary arteries	[2]
**Splendidofilariinae**	*Chandlerella chitwoodae* Anderson, 1961	Psittacopasserae, Galloanserae	Adults	Pulmonary arteries	[29]
	*Chandlerella bosei* (Chandler, 1924)	Corvidae, Certhidae, Muscicapidae	Adults	Whole lungs	[30]
	*Chandlerella sinensis* Li, 1933	Corvidae	Adults	Whole lungs and trachea	[27]
	*Dunnifilaria ramachandrani* Mullin et Balasingam, 1973	Muridae	Adults	Pulmonary arteries	[31]
	*Elaophora sagitta* (Linstow, 1907)	Bovidae	Adults	Pulmonary arteries	[32]
	*Paronchocerca ciconiarum* Peter, 1936	Ciconidae	Adults	Pulmonary arteries	[33]
	*Paronchocerca rousseloti* Chabaud & Biocca, 1951	Phasianidae	Adults	Pulmonary arteries	[33]
	*Paronchocerca francolina* (Jairajpuri and Siddiqi, 1970)	Phasianidae	Adults	Whole lungs, air sacs	[33]
	*Paronchocerca papillatus* (Ali, 1956)	Phasianidae	Adults	Whole lungs	[33]
	*Paronchocerca sonini Borgarenko*, 1984	Scolopacidae	Adults	Whole lungs	[33]
	*Paronchocerca struthionus* Bartlett and Anderson, 1986	Struthionidae	Adults	Whole lungs	[33]
	*Paronchocerca thapari* Deshmukh, 1969	Phasianidae	Adults	Whole lungs	[33]
	*Splendidofilaria algonquiensis* (Anderson, 1955)	Hirundinidae	Adults	Pulmonary arteries	[34]
	*Splendidofilaria caperata* Hibler, 1964	Corvidae	Adults	Pulmonary arteries (wall)	[35]
	*Splendidofilaria falconis* (Sonin, 1966)	Falconidae	Adults	Whole lungs	[30]
	*Splendidofilaria periarterialis* (Caballero, 1948)	Tyrannidae	Adults	Pulmonary arteries (wall)	[36]
**Setarinae**	*Setaria equina* (Abildgaard, 1789)	Equidae, Bovidae, Camelidae	Adults	Whole lungs	[37]
	*Setaria transcaucasica* Assadov, 1952	Bovidae, Cervidae	Adults	Whole lungs	[37]

First column indicates the filarial subfamily and second column the filarial species. Natural hosts are presented in columns 3 and experimental/accidental hosts are indicated in parenthesis. The filarial stage described in the lung is indicated in column 5; details on the pulmonary location are given in column 6. nd = not described in lungs.

## Materials and methods

### Ethics statement

All experimental procedures were carried out in strict accordance with the EU Directive 2010/63/UE and the relevant national legislation, namely the French “Décret No. 2013–118, 1er février 2013, Ministère de l’Agriculture, de l’Agroalimentaire et de la Forêt”. Protocols were approved by the ethical committee of the Museum National d’Histoire Naturelle (Comité Cuvier, Licence: 68–002) and by the “Direction départementale de la cohésion sociale et de la protection des populations” (DDCSPP) (No. C75-05-15).

### Parasites, mice and infection

*L*. *sigmodontis* was maintained in our laboratory, and infective third-stage larvae (L3) were recovered by dissection of the mite vector *Ornithonyssus bacoti* as previously described [38–40].

Six-week-old female BALB/c mice were purchased from Harlan (France) and maintained in the MNHN animal facilities on a 12-hours light/dark cycle. Infective L3 larvae were either inoculated or transmitted through the bite of the vector mite *O*. *bacoti* (“natural infection”). Mice were inoculated with 40 infective L3 either subcutaneously into the left lumbar area in 200 μl of RPMI 1640 or intravenously into the caudal vein in 50μl of RPMI 1640. For natural infections mites were left in contact with the mice for 12 h [40]. A group of 100 infected mites from the same batch was dissected under a binocular microscope to evaluate the average number of L3 per mite. Since it has been shown previously that no L3 remained in the blood-fed mites [40], this allowed us to evaluate the number of L3 given per mouse. Kinetics of infection were followed over 8 days of infection. Mice were sacrificed at 2 hours, 6 hours, 4 days, 6 days and 8 days post-inoculation (p.i.).

### Pleural lavage, pleural fluid isolation, pleural exudate cells and filarial load

The mice were anesthetized then sacrificed by final bleeding. The pleural cavity was washed 10 times with 1 ml of cold phosphate buffered saline (PBS) to collect pleural fluid, pleural exudate cells (PleCs) and filariae as previously described [41]. The first 2 ml were collected in a separate tube to limit pleural fluid dilution. The remaining 8 ml were isolated in a second tube. After 30 min deposition, the top 1 ml of the first tube was collected and centrifuged (5 min, 250g) then the pleural fluid supernatant was frozen (-20°C) for subsequent analyses. The PleCs pellet was taken up in the remaining 1 ml of the first tube and pooled into the 8 ml of the second tube. The filariae rapidly sediment at the bottom of the tube and the upper 8 ml containing the PleCs were transferred into a new tube and centrifuged (5 min, 250g). PleCs were diluted in 1ml medium and counted.

The isolated filariae were counted, analyzed by light microscopy (Olympus BX63 microscope, Olympus DP72 camera) and measured using the cellSens Dimension 1.9 software. The recovery rate of filariae, expressed as 100 x number of worms recovered/number of larvae inoculated (F/L3) was established.

Lungs were macroscopically examined after the pleural lavage and superficial petechiae were counted.

### Bronchoalveolar lavage (BAL)

The trachea was exposed and incised at the cervico-thoracic junction. A cannula was inserted and fixed with the thread. The bronchoalveolar space was washed with 10ml of cold PBS. The first ml was collected in a first tube, centrifuged (5 min, 250 g) and the supernatant (i.e. the BAL fluid) was frozen (-20°C). The pellet was pooled in the remaining 9 ml of the lavage. After centrifugation (5 min, 250 g), the pellet containing the BAL cells was diluted in 1ml of PBS– 2% foetal calf serum (FCS) (EUROBIO).

### Recovery of L3 in pulmonary lobes

Naïve, subcutaneously (SC) or intravenously (IV) infected mice were sacrificed by final bleeding at 2 hours, 6 hours, 4 days and 8 days post infection. After pleural and bronchoalveolar lavages with cold PBS, lungs were cut at the left and right principal bronchus to separate each pulmonary lobe and then removed. Right and left lung were placed separately in 5 cm Ø Petri dishes each containing 7 ml of PBS and torn up into small pieces (about 2–4 mm^2^). Petri dishes were examined under a binocular microscope from 1h up to 24h to allow the L3 to exit the tissue. Recovered L3 were counted and the recovery rate in lungs was established.

### Histology and immunohistology of the lung

Naïve, SC or IV infected mice were sacrificed 6 hours, 4 days and 8 days post infection. The lung was filled with and fixed in 4% formalin overnight. Fixative was changed 24 h post-fixation for a further 24 h. Thereafter, lungs were removed from the fixative and placed in 70% alcohol for 2–7 days before paraffin embedding. Five-micron-thick serial sections were prepared. For each lung, a hematoxylin-eosin (H&E) staining was performed. To characterize the peri-vascular space, a Masson's trichrome staining (Sigma-Aldrich) was performed to visualize collagen fibers according to the manufacturers' recommendations. Immunostained sections were firstly washed in PBS then their tissue’s peroxidase and biotin/avidin were blocked using dual endogenous enzyme block (Dako, France) and avidin/ biotin blocking kit (Vector, France) respectively. Neutrophils were stained with the primary antibody against Ly-6G/-6C (rat monoclonal Ab, clone NIMP-R14, Hycult Biotech) at 1/200 dilution, in blocking serum (Vectastain kit, Vector, France). Antigen retrieval was performed at pH 6 (Antigen unmasking solution, Vector, France). S100A9 was stained with the primary antibody against S100A9 (rat monoclonal Ab, clone MU14-2A5, Hycult Biotech) at 1/200 dilution, in blocking serum (Vectastain kit, Vector, France). Antigen retrieval was performed in a proteinase K solution (0.004%) diluted in a 1:1 glycerol-modified Tris Buffer (EDTA 3.7%, Triton X-100 0.5%, pH 8) incubated at 37°C for 10 min. Detection was performed using the Vectastain Elite ABC kit (Vector, France). Revelation was made with high sensitivity AEC substrate (Dako, France) then a quick counterstaining with Mayer’s haematoxylin (Merck, France).

### Precision cut lung slices (PCLS)

The mice were anesthetized then sacrificed by final bleeding. The thorax was opened and the trachea was exposed. The lung was filled via the trachea with 2% (w/v in PBS, pH 7.4) low melting point agarose warmed to 40°C (Sigma-Aldrich) in phosphate buffered saline. They were then removed from the thorax and transferred to ice cold PBS, then fixed 2h in cold 4% paraformaldehyde (PFA) in PBS and finally stored at 4°C in PBS. The left lung was isolated and was glued with the hilum facing downwards on cooled aluminium block using super glue. Then, 400 μm thick sections were cut using a vibratome (Microcut H1200 BioRad).

### Immunofluorescence

Precision cut lung slices were incubated with primary antibodies: rat anti-mouse Ly-6G (clone 1A8, Bio X-Cell), hamster anti-mouse CD31 (2H8, Life Technologies), rat anti-mouse S100A9 (MU14-2A5, Hycult Biotech), polyclonal rabbit anti-mouse Ci-H3 (ABCAM) diluted in PBS-BSA-Triton-for 48h at 4°C. The sections were washed in PBS and incubated with low species cross-reactive fluorophore-conjugated secondary antibodies (Cy3 / Cy5 anti-rat, anti-hamster, Jackson; AlexaFluor anti-rabbit Life Technologies) for two hours. Sections were washed in PBS-BSA-Triton, then PBS and post-fixed with 4% PFA for 5 min. After a subsequent rinsing step, the slices were transferred into a 24-well imaging plate (IBIDI), covered with buffered Mowiol 4–88, pH 8.5 (Sigma-Aldrich) then coverslipped. Sections were evaluated by using a confocal laser-scanning microscope (TCS-SP5, Leica).

### Analysis of cytokines and S100A9 in pleural and broncho-alveolar lavage fluids

Pleural and bronchoalveolar lavage (BAL) fluids were analyzed by ELISA for the content of IFN-γ, TNF-α, IL-10, IL-4, IL-1β, IL-6 and IL-17 ELISA kit (eBiosciences SAS, France), MCP-1 and CXCL1 ELISA kit (Peprotech, France), S100A9 and IL-33 (R&D, UK) following manufacturers’ guidelines. Results are expressed as pg/mL. Detection limits were 15 pg/ml for INF-γ and IL-33, 30 pg/ml for IL-10, 4 pg/ml for IL-4, IL-6 and IL-17, and 8 pg/mL for IL-1β, S100A9, CXCL1 and MCP-1.

### Cell analysis

PleCs and BAL cells were analyzed. Firstly, red blood cells were lysed by hypotonic shock. The cell suspensions were then centrifuged at 250 g for 8 min at 4°C, diluted in 1 ml PBS with 2% FCS and counted in PBS with 0.04% trypan blue by using a haemocytometer (KOVA Glasstic Slide). Cells were incubated 20 min with CD16/CD32. Proportions of the different leukocyte populations were determined by flow cytometry using the following rat anti-mouse antibodies: anti- F4/80-APC (dilution 1:200; eBioscience, clone BM8), anti-SiglecF-PE (dilution 1:200, BD Bioscience, clone E50-2440) and Ly6G-FITC (dilution 1:200, BD Bioscience, clone 1A8). Flow cytometry acquisition was performed using a FACSVerse flow cytometer running the FACSuite software (BD Biosciences). Doublets and debris were excluded. Analyses were performed with FACSuite Software.

### RNA extraction and reverse transcription

Naïve, SC or IV infected mice were sacrificed by final bleeding at 2 hours, 6 hours, 4 days and 8 days post infection. The lung was immersed in RNA later solution (Ambion, France) and then frozen at -80°C before extraction. Total RNA was extracted using an RNeasy mini kit (Qiagen, Germany), according to the manufacturer’s instructions. A DNase (Invitrogen, France) treatment was performed to eliminate remaining DNA. Reverse transcription was performed using non-specific oligo p(dT) (Roche Diagnostics, France) and SuperScript III reverse transcriptase (Invitrogen, France).

### Lung s100a8/9 expression analysis

Real-time PCR gene-specific primers for s100a8, s100a9 and β-actin were designed using Oligo Calc (Kibbe, 2007) as follow: s100a8, 5’-ACCATGCCCTCTACAAGAA TGACT-3’; 5’-ACTCCTTGTGGCTGTCTTTGTG-3’; s100a9, 5’-AACCAGGACAATCAG CTGAGCTTT-3’; 5’-AGGCCATTGAGTAAGCCATTCCC-3’; β-actin, 5’-ACCACAGCTGAGAGGGAAATCGT-3’; 5’-AACCGCTCGTTGCCAATAGTGA-3’. Real-time PCR was performed using the DNA Master Plus SYBR Green Kit (Roche Diagnostics, France) in a LightCycler 2.0 (Roche Diagnostics, France) with an initial incubation of 10 min at 95°C, 40 amplification cycles of ten seconds at 95°C, of eight seconds at 60°C, and of ten seconds at 72°C, during which the fluorescence data were collected. This program was followed by a step of fusion. The 10 μL reaction mixture contained 1X DNA MasterPlus SYBR Green (QIAGEN, France), 0.5 μM of each primer, and 4 μL of template. s100a8 and s100a9 gene expression was determined relative to β-actin using the 2^-ΔΔCT^ method.

### Lung cytokine expression analysis

A cytokine array (Mouse Cytokines & Chemokines RT^2^ Profiler PCR Array, Qiagen, Germany) was performed on a pool of cDNA from 8 naive or 8 D4 subcutaneously infected mice according to the manufacturer’s instructions. The array comprises 84 probes for secreted cytokines. The arrays were scanned with a 7300 Real-Time PCR System (Applied biosystem). Data was processed and displayed using the online RT2 Profiler PCR Array Data analysis 3.5 software at the sabiociences.com website (Qiagen). Gene expression was normalized to 5 housekeeping genes (Actb, B2m, Gapdh, Gusb, Hsp90ab1). Transcripts with fold change >2 were selected. Transcriptional data were evaluated using Ingenuity Pathway Analysis (IPA, Systems Inc., USA) and prediction (increase of decrease) of biological activities occurring in the tissue was established. Validation was performed by qRT-PCR for CXCL1 on individual samples from SC- and IV-infected mice at day 4 p.i. Specific primers for CXCL1 were designed using Oligo Calc (Kibbe, 2007) as follow: 5’- CACTGCACCCAAACCGAAGTCATA-3’; 5’-TCTCCGTTACTTGGGGACACCTTT -3’; A DNA Master Plus SYBR Green Kit (Roche Diagnostics, France) was used in a LightCycler 2.0 (Roche Diagnostics, France) with an initial incubation of 10 min at 95°C, 40 amplification cycles of ten seconds at 95°C, of 8 sec at 60°C, and of 10 sec at 72°C, during which the fluorescence data were collected. This program was followed by a step of fusion. The 10 μL reaction mixture contained 1X DNA MasterPlus SYBR Green (QIAGEN, France), 0.5 μM of each primer, and 5 μL of template. CXCL1 gene expression was determined relative to β-actin using the 2^-ΔΔCT^ method.

### Statistical analyses

The choice of statistical tests was based on sample size, normality (Shapiro-Wilk test) and homoscedasticity (Bartlett’s test), examined prior to further analysis. Data from independent experiments were pooled when possible. When normality was established, results were analyzed by t-test, one-way ANOVA test in order to determine the effect of one factor, i.e., the group of mice, or two-way ANOVA in order to determine the effects of two factors, i.e., the group of mice and the time, or their interaction followed by a Bonferroni’s multiple comparisons post-tests; otherwise non-parametric Kruskal Wallis test followed by a Dunn’s multiple comparisons post-test was used. Representation and data analyses were performed with GraphPad Prism 5 software.

## Results

### 1. Migration of infective larvae to the pleural cavity: Presence in the lung and comparison between different modes of inoculation

**Fig 1:**
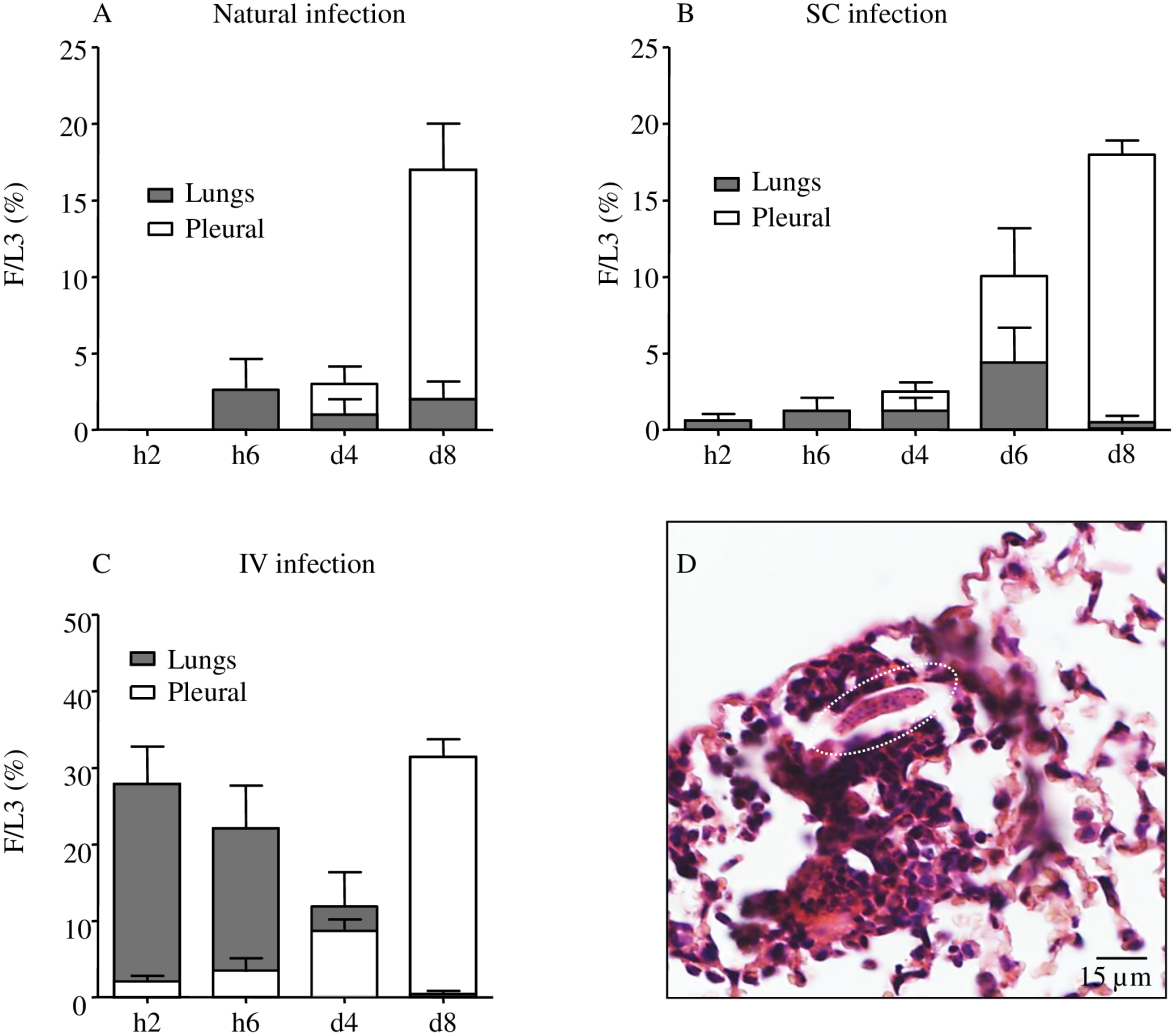
L3 presence and loads in the lung and in the pleural cavity. BALB/c mice were inoculated with 40 L3 of *L*. *sigmodontis* either subcutaneously (SC) or intravenously (IV) or L3 were transmitted through the bite of the vector mite *O*. *bacoti* (“natural infection”). (A-C) *L*. *sigmodontis* recovery rate (F/L3) on hour 2 (h2), hour 6 (h6), day 4 (d4) and day 8 (d8), once L3 were recovered either in the mechanically disrupted lungs or in the pleural cavity and counted. (A) Recovery rate in the lung (grey bars) and pleural cavity (white bars) of naturally infected mice (n = 8 per time point, pool of 2 independant experiments) (B) Recovery rate in the lung and pleural cavity of SC infected mice. (h2: n = 8, pool of 2 independent experiments); h6-d4-d8: n = 19–24, pool of 5 independent experiments; d6: n = 7). (C) Recovery rate in the lung and pleural cavity of IV infected mice. h2-h6, n = 8–12 (pool of 2 independent experiments); d4-d8, n = 6–8 (lung, pool of 2 independent experiments), n = 24 (pleural, pool of 5 independent experiments). (d) Haematoxylin-Eosin staining of lung sections at 6 hours post inoculation showing one L3 in lung tissue (white dotted circle). Bars represent the mean ± SEM.

To define the kinetics of arrival of L3 in the pleural cavity, we analyzed the L3 content of both mechanically disrupted lungs and pleural cavity of mice in which L3 had either been transmitted through the bite of the vector mite *O*. *bacoti* (“natural infection”) or subcutaneously (SC) injected, at various time points from 2 hours (h2) to 8 days (d8) p.i. (Fig 1A and 1B).

A small number of L3 were detected h2, h6 and d4 p.i. in the pleural cavity of the SC- or naturally-infected mice (recovery rate < 5%). Between d4 and d8 the number of L3 in the pleural cavity increased (recovery rate up to almost 20%) (Fig 1A and 1B).

We also observed L3 in the lung of infected mice (natural infection or SC inoculation) (Fig 1A and 1B). These results are in concordance with earlier observations by Wenk and Bain, who had noticed the presence of *L*. *sigmodontis* L3 in mechanically disrupted lungs of rodents [2,11,12,42,43] (S1 Fig). Taken together these data show the progression of L3 from skin to pleural cavity (S1 Fig). L3 disappear from the skin within a couple of days. They are observed in the lymphatics hours to days post infection peaking at d2 and d3 p.i, before reaching the pleural cavity and accumulating there between d4 and d6 p.i. Once in the pleural cavity the recovery rate remains stable for a period of time depending on the rodent host species, from 10 to 30 days p.i. for SC-infected BALB/c mice as reported in [5,12,44]. Hence the filarial load is determined after the migration phase of the larvae by counting the number of parasites in the pleural cavity.

The presence of L3 in the lung was observed throughout the migration phase peaking at d6 p.i (Fig 1B). The number of mice exhibiting L3 in the lung increased over the time from 25% at h2 p.i. up to 85% at d6 p.i.

The most parsimonious way to explain the presence of L3 in the lung would be that L3 follow the lymphatic flow and thus reach the blood pulmonary circulation, as the lymphatic flow merges into the blood pulmonary circulation at the level of the thoracic duct.

In order to bypass the skin and the lymphatic migration phase and thus to limit the asynchronic arrival of L3 in the pleural cavity we inoculated L3 intravenously. This is the first time that this kind of delivery has been tested with *L*. *sigmodontis* L3 in rodents. L3 not only survived to this mode of inoculation but strikingly, the recovery rate of L3 was higher in the pleural cavity (Fig 1C), almost twice as much as the one observed in SC- or naturally-infected mice at the same time point. Larvae were observed in the pleural cavity as early as h2 p.i., accumulating regularly up to d8 p.i (around 2% at h2, 4% at h6 and 10% at d4). During this time frame we clearly observed a balance between lungs and pleural cavity: recovery rate in the lungs peaked as early as h2 p.i, decreasing then to d8 p.i. (around 30% at h2, 26% at h6, 13% at d4 and below 1% at d8).

**Fig 2:**
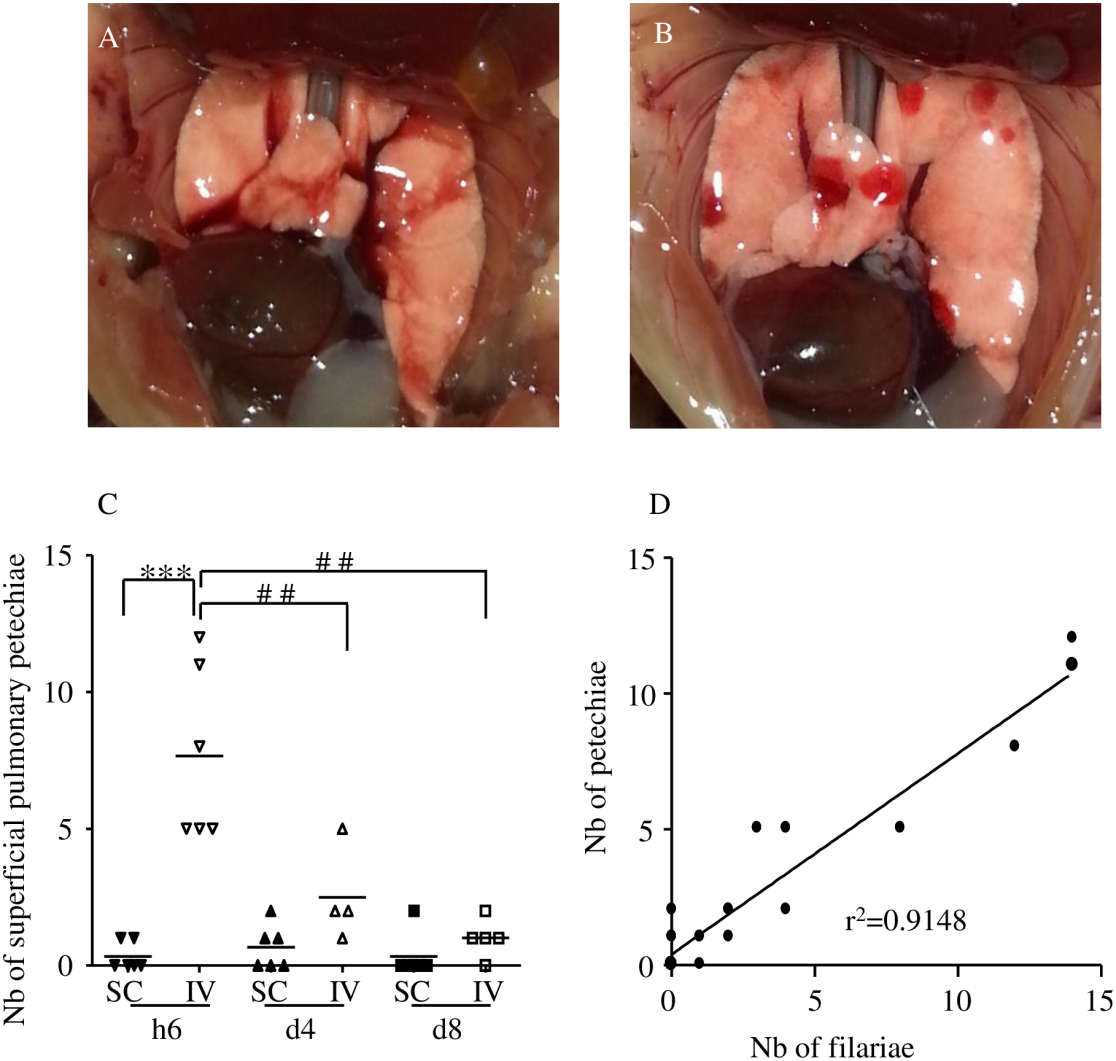
Hemorrhages in the lung of infected mice. BALB/c mice were inoculated with 40 L3 of *L*. *sigmodontis* either subcutaneously (SC) or intravenously (IV). Representative picture of (A) a normal lung, (B) a lung with superficial numerous roundish well-delineated red hemorrhages. (C) Number of superficial pulmonary hemorrhages in lungs at six hours (h6), four days (d4) and eight days (d8) post inoculation. n = 6, bars represent the mean ± SEM; two-way ANOVA followed by Bonferonni, *** = *p*<0.001 (difference between IV- and SC-infected mice), ^##^ = *p*<0.01(difference between timepoints in IV-infected mice). (D) Correlation test (Pearson) between the number of L3 recovered in the lung and the number of hemorrhages, r^2^ = 0.9148.

**Fig 3:**
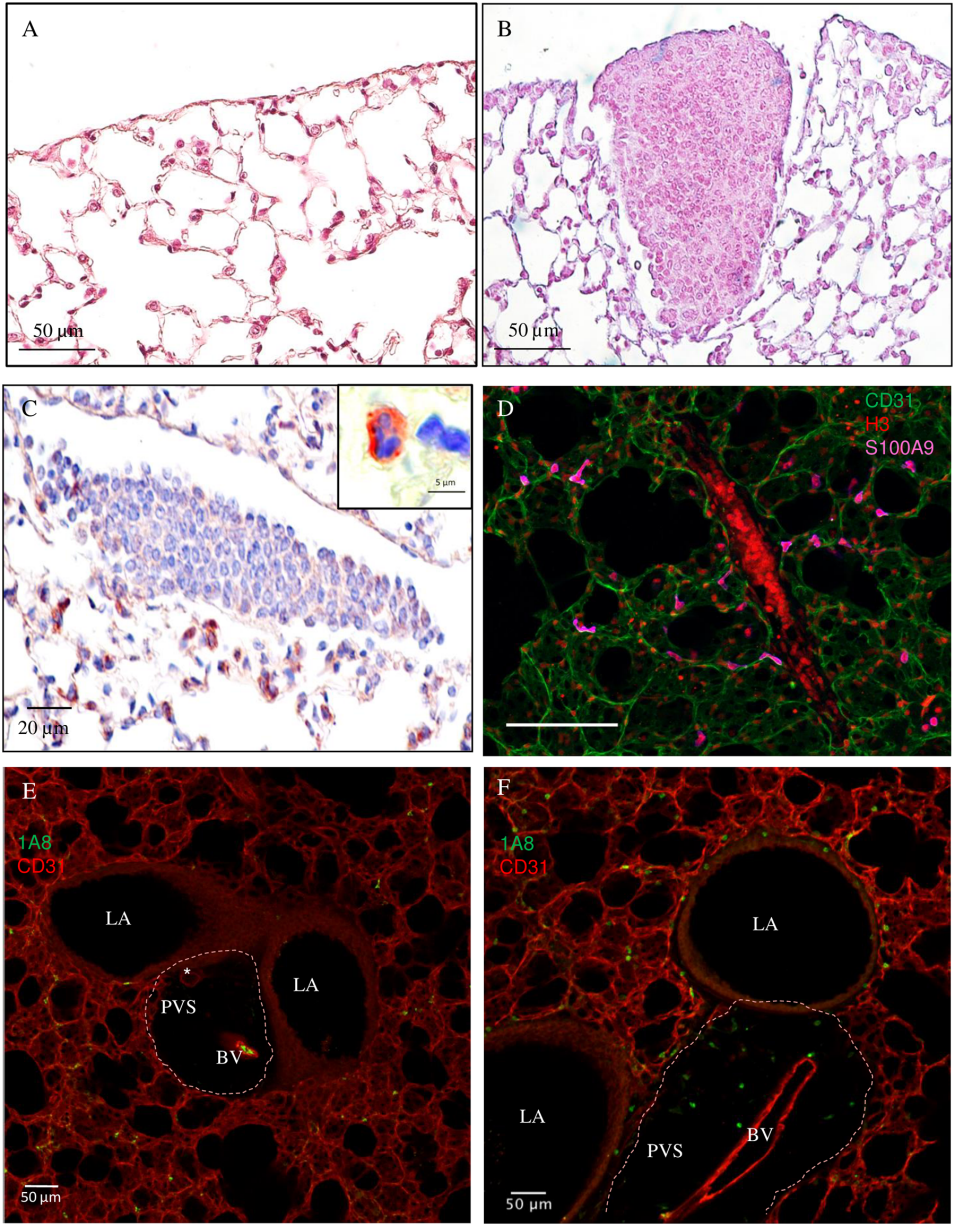
Granulomas and neutrophil-infiltrated peri-vascular space in the lung of infected mice. BALB/c mice were inoculated with 40 L3 of *L*. *sigmodontis* either subcutaneously (SC) or intravenously (IV). After two hours (h2) six hours (h6), two days (d2), four days (d4) and 8 days (d8) post inoculation, lung sections were prepared. (A) Haematoxylin-Eosin staining of a naïve lung section showing normal parenchyma and mesothelium. (B) Haematoxylin-Eosin staining of a lung section showing a granuloma in SC-infected mice at d8 p.i. (C) Ly6G/C (clone NIMPR-R14) immunostaining of lung sections at d8 p.i. (from a SC-infected mouse) were performed showing absence of neutrophils within the granuloma but presence in the surrounding tissue. Neutrophils were differentiated from monocytes by their nuclei shapes (cf corner zoom) (D) Representative maximum intensity projection from a confocal z-stack of S100A9 (an abundant neutrophil protein, magenta) immunostaining of lung precision cut lung slices (PCLS) at d8 p.i. (IV-infected mouse) showing absence of neutrophils within the granuloma but presence in the surrounding tissue; CD31 (green) stain for endothelial cells and histone H3 (red) for cells. (E-F) Representative maximum intensity projection from a confocal z-stack of a lung PCLS with CD31^+^ capillaries (red) surrounding larger airways (LA) and a peri-vascular space (PVS) (the boundaries of which are marked by dashed line) containing a blood vessel (BV) and a lymphatic vessel (*) with (E) the absence of Ly6G+ (clone 1A8) neutrophils (green) in the PVS of naïve mouse; and (F) the presence of Ly6G+ neutrophilic infiltrates in the PVS and around airways in a d4 IV-infected mouse.

In both models (delivered in the skin *versus* intravenously) pulmonary locations suggest that L3 have exited the lung capillaries (as suggested by Wenk [11]) probably migrating through the pulmonary parenchyma then though the visceral mesothelia to arrive in the pleural cavity. We observed the presence of L3 inside the lung (Fig 1D), which is associated with the recruitment of cells including numerous polymorphonuclear neutrophils around the larvae, as well as various damage in the lung of infected mice (Figs 2 and 3) supporting a disruptive transpulmonary migration.

### 2. The passage of L3 through the lung induces inflammation characterized by local haemorrhages, granulomas and neutrophil accumulation in the perivascular spaces

During necropsy of infected mice, petechiae were recurrently noticed on the surface of the lung in IV-inoculated mice (Fig 2B, compared to Fig 2A). Therefore, this phenomenon was quantified at h6, d4 and d8 p.i. (Fig 2C). Small haemorrhagic areas were rarely observed on the lung of SC-infected mice whereas the lung of all the IV-infected mice exhibited a high number of such areas at both h6, these decreased at d4 p.i and even more d8 p.i A clear correlation (r^2^ = 0.9148) was observed between the number of petechiae and the number of recovered L3, independently of the mode of inoculation and the time of infection (Fig 2D).

As we previously observed [13], granulomas were found at d8 p.i. in the lung of 50% of SC-infected mice. Such granulomas were also observed in IV-infected mice at d8 p.i and in both SC- and IV-infected mice at d4 p.i. but with lower prevalence (Fig 3A–3D). At these time points, granulomas mainly consisted of F4/80^+^ macrophages and CD3^+^ lymphocytes. Neutrophils were absent as evidenced by the lack of Ly6G/C positive polymorphonucleic neutrophils (Fig 3C) and the lack of S100A9 neutrophils (Fig 3D).

However, neutrophils were present in higher number in specialized areas of the lung. Lymphatic and blood CD31+ capillaries formed a meshwork within the connective tissue surrounding respiratory bronchioles and larger airways. Larger blood vessels and lymphatic vessels can be found close to large airways in a perivascular space containing collagen fibers [45]. 1A8^+^ neutrophilic infiltrates were observed in these perivascular spaces only in infected mice at later time points (d4 and d8 p.i.), independently of the mode of inoculation of L3 (Fig 3E and 3F).

**Fig 4:**
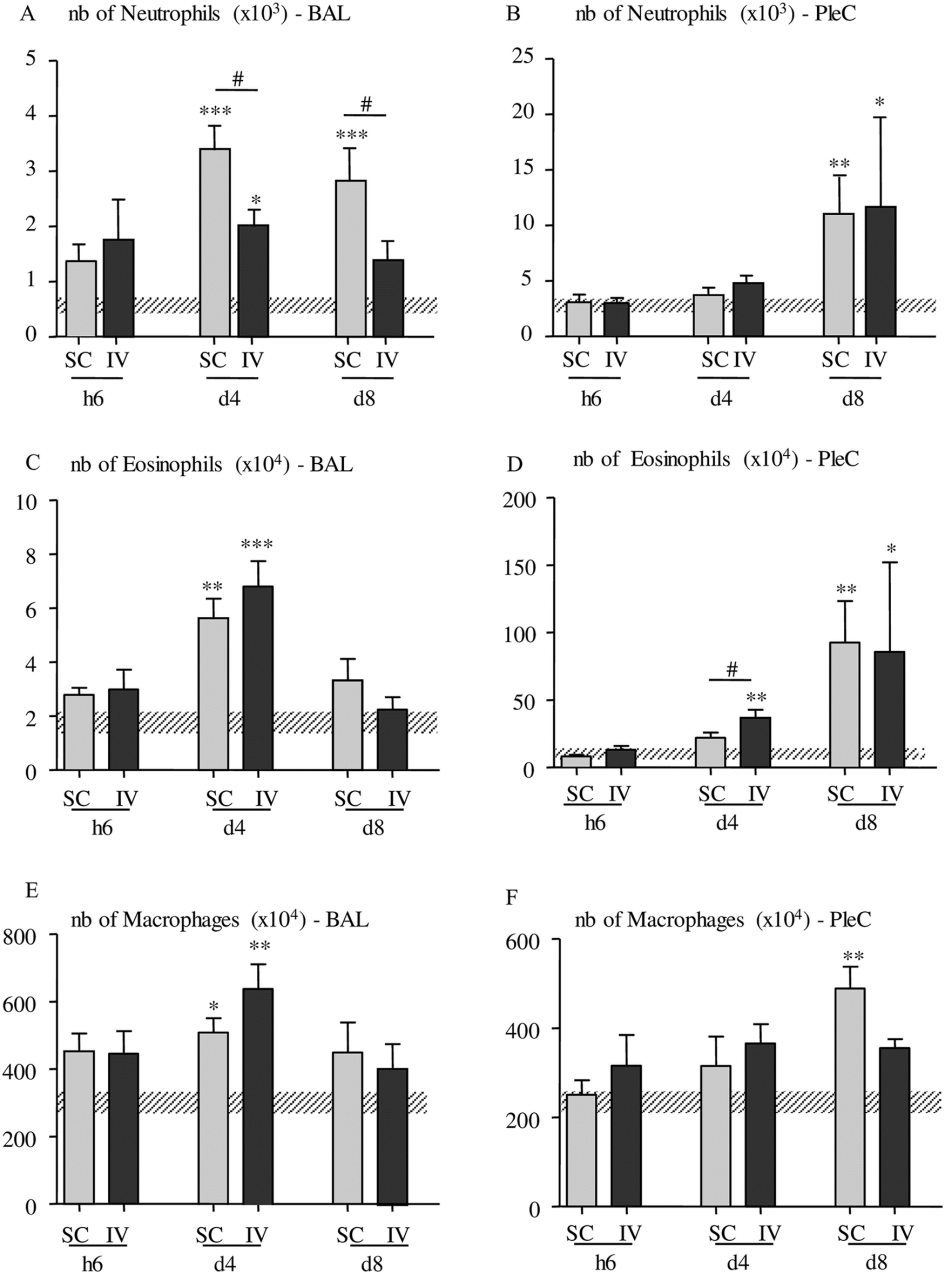
Quantification of macrophages, eosinophils and neutrophils in the bronchoalveolar and pleural spaces. Flow cytometry phenotypic analyses of Ly6G neutrophils (clone 1A8), F4/80 macrophages (clone BM8) and Siglec F eosinophils (clone E50-2440) were performed at different time points (h2, h6, d4 and d8) post inoculation on pleural cells (PleC) or cells isolated from broncho-alveolar lavage (BAL) from IV- and SC-infected mice. (A-B) Number (nb) of F4/80+ macrophages in the broncho-alveolar lavage (A) and in the pleural cells (B); (C-D) Number of Siglec F+ eosinophils in broncho-alveolar lavage (C) and in the pleural cells (D);.(E-F) Number of Ly6G+ neutrophils in the broncho-alveolar lavage (E) and in the pleural cells (F). Number of cells in uninfected mice are represented by a dashed horizontal bar. The results are expressed as mean ± SEM, n = 4–6, Kruskal-Wallis followed by a Dunns (*p<0.05. **p<0.01, ***p<0.001 between infected and naive mice, #p<0.05 difference between SC- and IV-infected mice for a given time point).

An increase in neutrophils was also noticed in the cells from the bronchoalveolar lavage (Fig 4A) at d4 and d8 p.i in SC-inoculated mice and to a lesser extent only at d4 p.i. in IV-inoculated mice. Neutrophil numbers increased in the pleural cavity at d8 p.i. in both SC- and IV- inoculated mice (Fig 4B).

Similarly, at these time points (d4 and/or d8 p.i.) an increase in eosinophils and macrophages was both noticed in the bronchoalveolar lavage (Fig 4C and 4E) and the pleural cavity (Fig 4D and 4F).

### 3. Increase of S100A9^+^ neutrophils in the lung

**Fig 5:**
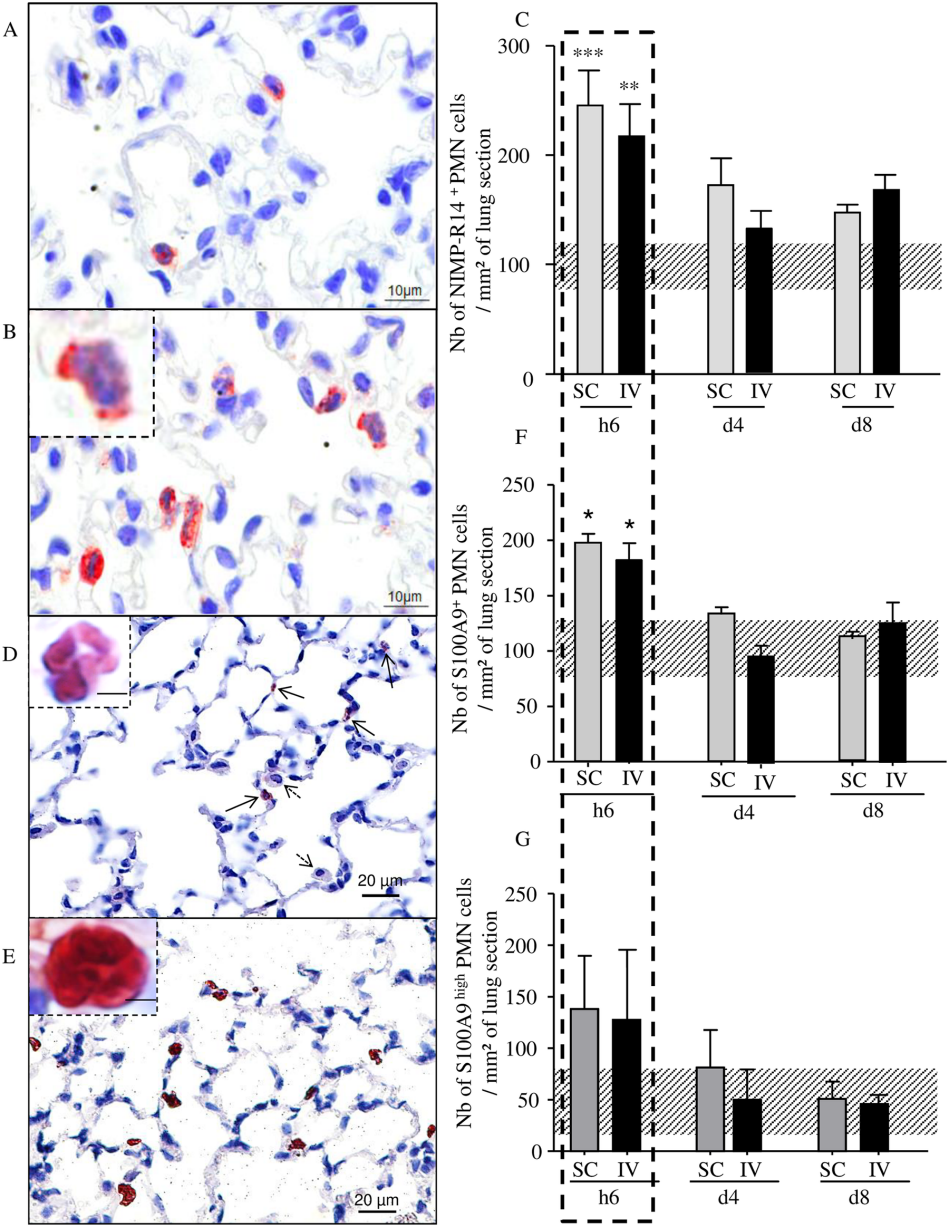
Transient early increase of S100A9 expressing neutrophils in the lung of infected mice. BALB/c mice were inoculated with 40 L3 of *L*. *sigmodontis* either subcutaneously (SC) or intravenously (IV). Lung sections were analysed six hours (h6), four days (d4) and 8 days (d8) post inoculation. (A) Ly6G/C (clone NIMPR-R14) immunostaining of lung sections were performed showing a few interstitial neutrophils in naive mice (A) and an increase of neutrophils in h6-infected mice (B). Neutrophils were differentiated from monocytes by their nuclei shapes (B, corner zoom). (C) Number of neutrophils per mm^2^ on Ly6G/C immunostained sections; bars represent mean ± SEM, n = 4–6 (pool of 3 independent experiments), two-way ANOVA followed by a Bonferonni (**p<0.01, ***p<0.001). Uninfected mice are represented by a dashed horizontal bar. (D-G) S100A9 (clone MU14-2A5) immunostaining of lung sections were performed showing (D) low staining of S100A9 in neutrophils (black arrows). Alveolar macrophages (dotted arrows) are S100A9^-^. Top left corner: zoom on S100A9^low^ neutrophil, (E) high staining of S100A9 in neutrophils. Top left corner: zoom on S100A9^high^ neutrophil. (F) Total number of S100A9^+^ neutrophils and (G) number of S100A9^high^ neutrophils per mm^2^ of lung. Uninfected mice are represented by a dashed horizontal bar. Results are expressed as mean ± SEM; n = 4 mice, 2–3 slides per mouse and 2–4 sections per slide were analyzed.

Although the presence of neutrophils in perivascular spaces was observed days p.i., neutrophils surrounding L3 were observed hours p.i. in the pulmonary parenchyma (Fig 1E). Within the first hours of the filarial infection, neutrophils were observed inside the lung tissue, most often in the pulmonary capillaries and more rarely in the alveolar space (Fig 5A and 5B). Scoring neutrophils revealed a transient increase number of these cells on lung sections at h6 p.i. in both SC and IV infected mice (Fig 5C).

We then checked for S100A9 and S100A8, small calcium-binding proteins that are found at high levels in the extracellular medium during inflammatory conditions. Immunostaining of lung sections revealed that all S100A9 positive cells were morphologically identified as neutrophils (Fig 5D and 5E) and a large percentage of neutrophils were also S100A9^+^ (Fig 5F). We distinguished two states of S100A9^+^ neutrophils according to their content in S100A9, either low (Fig 5D, zoom) or high (Fig 5E, zoom). Scores of S100A9+ neutrophils (Fig 5F) and S100A9^high^ neutrophils (Fig 5G) were established revealing a peak of S100A9^+^ neutrophils at h6 p.i. in both SC- and IV-infected mice with a majority of S100A9 high neutrophils (74% and 70% respectively).

### 4. Increase in S100A9 protein in both bronchoalveolar and pleural fluids and in s100a8/9 transcription in the lung

**Fig 6:**
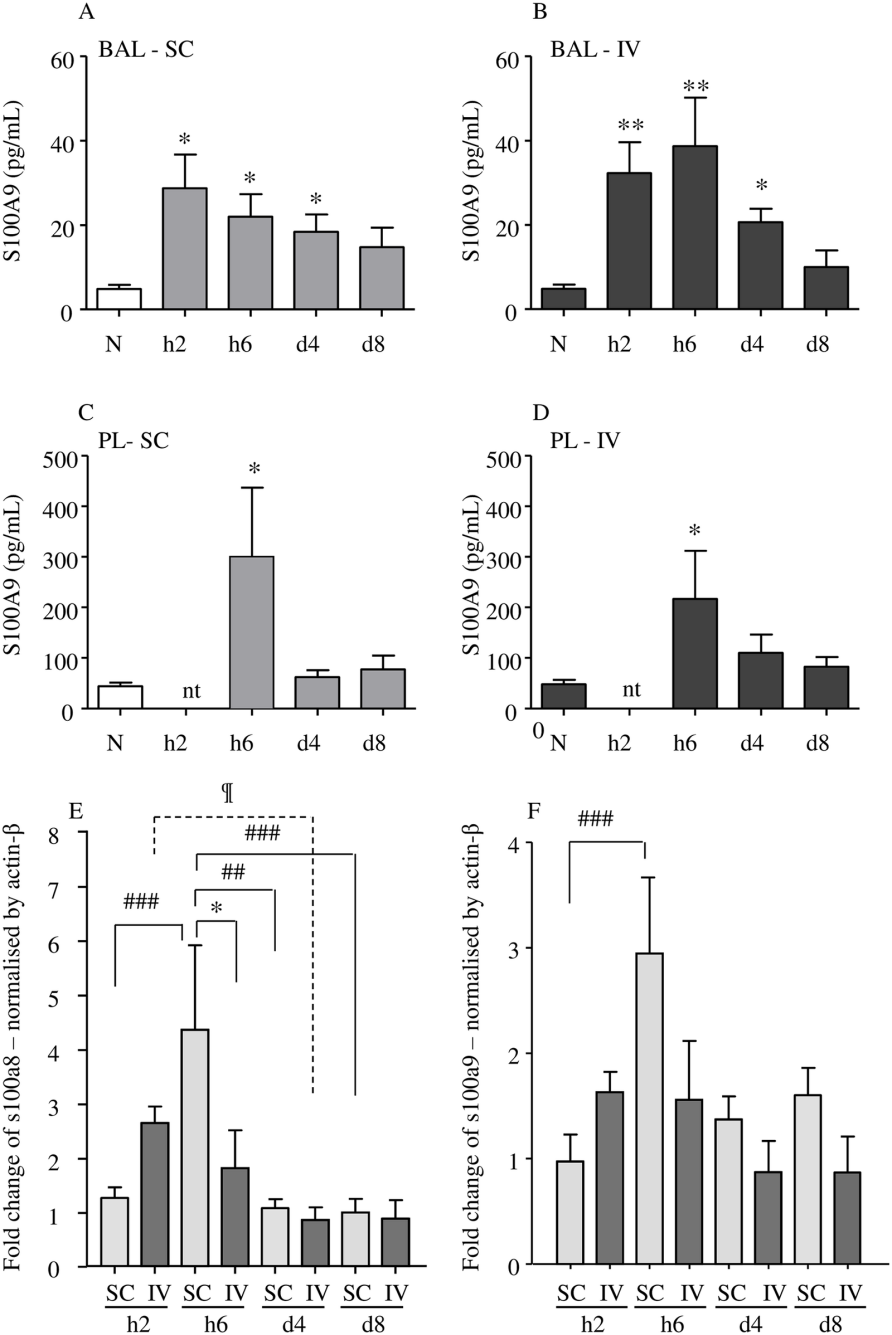
Transient early increase of S100A9 in bronchoalveolar and pleural fluids and of s100a8 / s100a9 transcripts in lungs. BALB/c mice were inoculated with 40 L3 *L*. *sigmodontis* either subcutaneously (SC) or intravenously (IV). Two hours (h2), six hours (h6), four days (d4) and 8 days (d8) post inoculation, mice were sacrificed. Bronchoalveolar and pleural lavages were performed then lungs were isolated and frozen. (A—D) Bronchoalveolar fluid (BAL) (A & B, respectively SC and IV infected mice; n = 6) and pleural fluid (PL) (C & D, respectively SC and IV infected mice; n = 10–12, pool of 3 independent experiments) were tested for S100A9 by ELISA. The results are expressed as mean ± SEM. One way ANOVA followed by a Bonferonni, ** = *p*<0.01, * = *p*<0.05 (difference between infected and naïve mice). nt: not tested. (E-F) A q-RTPCR was performed for (E) s100a8 and (F) s100a9 transcripts. Normalization was made with β-actin housekeeping gene by 2^-ΔΔCT^ method, n = 5–6 (pool of 3 independent experiments). The results are expressed as fold-change mean ± SEM; a two-way ANOVA followed by a Bonferonni was performed, *p<0.05 difference between IV-and SC- infected mice, ^##^p<0.01, ^###^p<0.001 difference between timepoints.

To further analyze the S100A9 response in the lung, the S100A9 protein level was determined in both bronchoalveolar and pleural fluids (Fig 6A–6D). The protein was similarly increased at h6 p.i. in both IV and SC infected mice in both fluids. However, the levels were much higher in pleural than bronchoalveolar fluids.

We also evaluated the level of s100a9 and s100a8 transcription at h2, h6, d4 and d8 p.i. in lung tissue. Transcripts were increased after a few hours p.i. before decreasing over days in either SC- or IV- infected mice (Figs 6E and 5F). Peaks were detected earlier for IV-inoculated mice (h2 p.i. instead of h6 p.i. for SC-inoculated mice), but the level of the peaks was slightly higher in SC-inoculated mice.

### 5. A proinflammatory cytokine/chemokine response is mounted hours after filarial infection followed within days by a process of regulation

**Fig 7:**
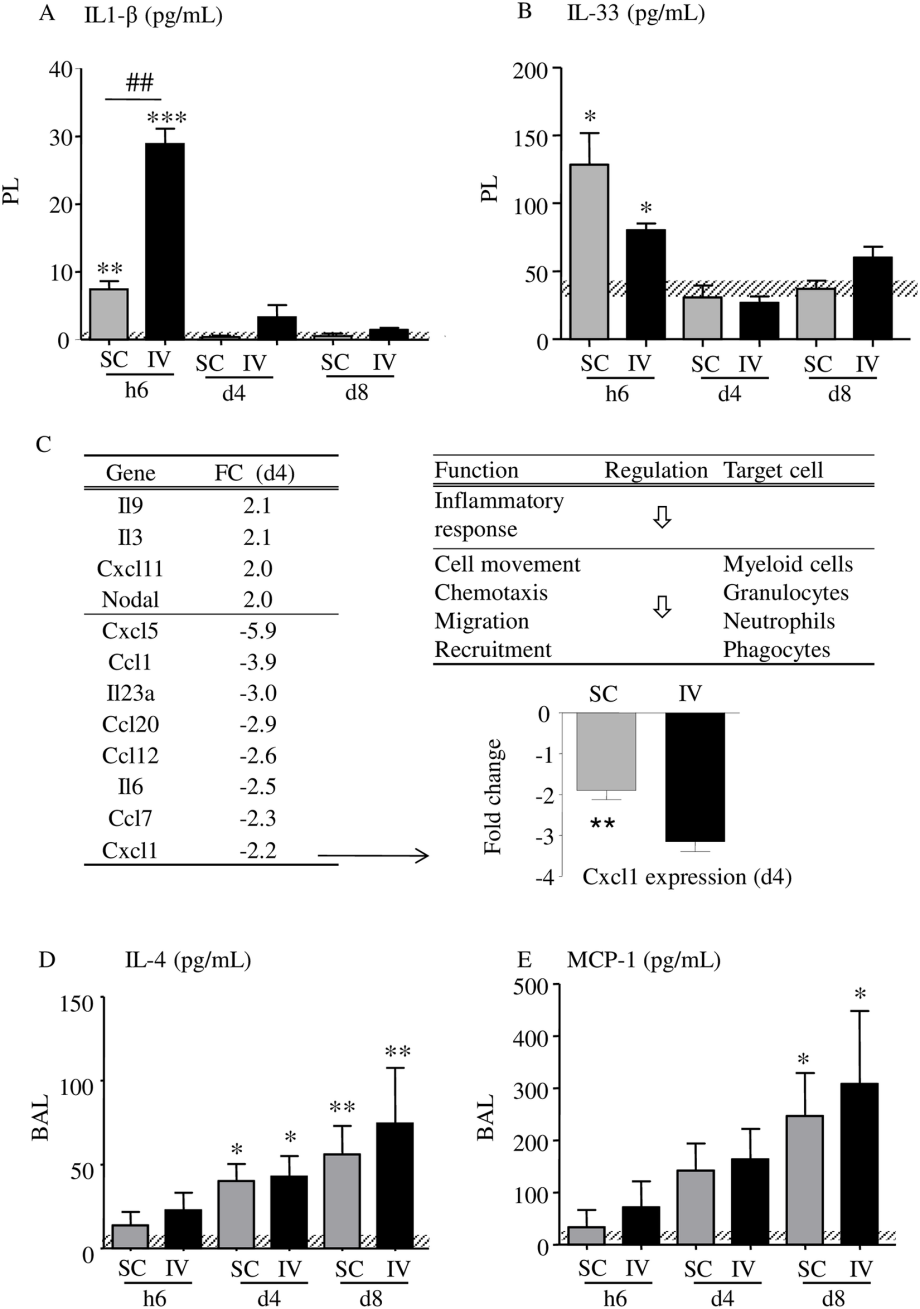
Inflammation and regulation. Dosage of IL-1β (A) and IL-33 (B) in the pleural fluid (PL) by ELISA, n = 4–8 (pool of 2 independent experiments). Uninfected mice are represented by a dashed horizontal bar. (C) Cytokines/chemokines transcripts (cut-off: >2-fold change, FC) induced in the lung of SC-infected mice at d4 p.i and associated functions. The effects of the gene expression changes in the lung were predicted using Ingenuity Pathways Analysis (IPA); regulation of the functions is indicated by arrows. (D) A q-RTPCR was performed for Cxcl1 transcripts to validate the array results; n = 12 (SC) and 5 (IV). Dosage of IL-4 (D) and MCP-1 (E) in the broncho-alveolar lavage (BAL) by ELISA, n = 5–6. Uninfected mice are represented by a dashed horizontal bar. The results are expressed as mean ± SEM; a two-way ANOVA followed by a Bonferonni was performed for A, B, D and E, *p<0.05, **p<0.01, ***p<0.001 differences between infected mice and naive, ##p<0.01 difference between SC- and IV-infected mice at h6 for IL-1 β; a t-test was performed for Cxcl1, **p<0.01.

Our results suggest two different responses through the migratory phase of L3, an early one, which takes place within the first hours after infection, and a later one, which is seen within days after infection. In addition to the proinflammatory protein S100A9, different cytokines known to be involved in inflammation or T helper cell pathways were tested in both pleural and bronchoalveolar fluids collected at h6, d4 and d8 p.i. in mice (Fig 7 and Table 2). In the pleural fluids, IL-1β and IL-33 increased at h6 p.i. in both SC and IV infected mice (Fig 7A) supporting an early inflammation likely due to the initial lung crossing by L3. These cytokines were not detected in the BAL fluid.

**Table 2:** Cytokine overview.

Cytokine	Injection	h6	d4	d8	h6	d4	d8
IL-1β	SC	0	0	0	**+**	=	=
	IV	0	0	0	**+++**	=	=
IL-4	SC	**+**	**++**	**++**	=	=	=
	IV	**+**	**++**	**++**	=	=	=
IL-6	SC	0	0	0	0	0	0
	IV	0	0	0	0	0	0
IL-10	SC	=	=	=	=	=	=
	IV	=	=	=	=	=	=
IL-17A	SC	=	=	=	=	=	=
	IV	=	=	=	=	=	=
IFN-γ	SC	=	=	=	=	=	=
	IV	=	=	=	=	=	=
MCP-1	SC	**+**	**++**	**++**	=	=	=
	IV	**+**	**++**	**++**	=	+	=
TNF-α	SC	=	=	=	=	=	=
	IV	=	=	=	=	=	=
IL-33	SC	0	0	0	**+**	=	=
	IV	0	0	0	**+**	=	=
CXCL1	SC	=	=	=	**=**	=	**++**
	IV	=	=	=	**=**	=	**++**

BALB/c mice were inoculated with 40 L3 of *L*. *sigmodontis* either subcutaneously (SC) or intravenously (IV). Six hours (h6), four days (d4) and 8 days (d8) post inoculation, mice were sacrificed and broncho-alveolar and pleural washes were performed. ELISAs were realized in the broncho-alveolar fluid (BALF) and the pleural liquid (LP). First column indicates the tested cytokines. The second column gives the mode of L3 inoculation in mice (SC or IV). The third and fourth columns show the results for the BALF and the LP respectively. These two columns are subdivided in three sub-columns according to the considered time point (h6, d4 or d8). Scores were as follows: 0, no detection; =, detected but no difference between uninfected and infected groups; +, detection (from low + to high +++). n = 5–6 for BALF (pool of 2 to 3 independent experiments); n = 10–12 for LP (pool of 2 to 3 independent experiments).

To further characterize the inflammatory environment in the second phase i.e. days post infection, cytokine/chemokine transcripts were analysed in mouse lungs at d4 p.i. (Fig 7C) showing a strong down regulation of the inflammatory response and functions such as chemotaxis and recruitment of neutrophils and phagocytes. For example, CXCL1, a potent chemoattractant for neutrophils, is down regulated in both SC- and IV- inoculated mice in the lung tissue.

The levels of IL-4 and MCP-1, involved in the recruitment of eosinophils and macrophages respectively, also increased significantly later during the infection at d4 and/or to d8 p.i. and their production was correlated. By acting on macrophages which are critically involved in inflammation, IL-4 and MCP-1 could induce alternative macrophage activation promoting tissue repair. CXCL1 was increased in the pleural fluid at d8 (Table 2).

## Discussion

Within 8 days the infective larvae migrate from the cutaneous inoculation site via the lymphatic drainage through the heart and lung into the pleural cavity. There *L*. *sigmodontis* L3 moult into L4 around day 9–10 post-inoculation in the pleural cavity of BALB/c mice, slightly earlier in jirds or cotton rats [1,15].

Due to their complex route of migration, the L3 have to cross various anatomical bottlenecks resulting in a loss of synchronicity concerning their arrival inside the pleural cavity where they accumulate over the days until day 6–8 post infection, depending on the rodent host species. Hence the filarial load is already determined after the migration phase of the larvae.

Whether L3 are transmitted through the bite of the haematophagous vector or subcutaneously injected, most of the incoming L3 are counteracted in the skin of the rodent. It was suggested that the successful L3 could escape the inflammatory response of the skin by entering the afferent lymphatic system [2]. Once in lymphatics, L3 are distributed through afferent lymphatic vessels [2,46], there they have to go beyond the draining lymph nodes to reach the efferent lymphatic vessels. Disrupting lymph nodes at various time points from rodents infected by *L*. *sigmodontis* revealed the continuous presence of L3 in the lymphatic system from hours to days post infection, with a peak at d2 and d3 (S1 Fig) [2,11,12,42,43]. Understanding the passage of *L*. *sigmodontis* L3 to the pleural cavity requires to take into account the cardiopulmonary system, firstly because of its anatomy and secondly because of necropsy results. The latter would be consistent with previous studies [2,12] revealing the presence of L3 in the cardiopulmonary compartment hours to days after their subcutaneous inoculation, although it was not possible to confirm their specific presence in the lung rather than in the heart. Analyzing the lung only has allowed us to demonstrate the presence of L3 in this tissue (Fig 1). How to explain the presence of L3 in the lung? The structure of bicuspid valves in the lymphatic vessels makes it difficult to move backwards. Valves acts as unidirectional gates for filaria migrating within the lymphatic vessels. The lymphatic vessels merge into the thoracic duct and the right lymphatic duct that are drained into the subclavian veins. After passage through the right ventricle of the heart, the blood and therefore the L3 are drained into the pulmonary arteries then into the pulmonary capillaries irrigating the lung, which are only 10 μm in diameter [47]. L3 are large organisms (750–800 μm length, 10–12 μm diameter) displaying a powerful musculature [48] allowing their motility. They can also release excreted/secreted molecules [49], which might facilitate their passage from the pulmonary capillaries through the lung to the pleural cavity. It is therefore possible, that a part of the inoculated L3 either get trapped in the lung and is unable to make their way to the pleural cavity, or that some L3 remain in the blood and reach the general blood circulation. These L3 could then be destroyed in the organs of clearance such as the liver or the spleen which are both highly vascularized.

To bypass the skin and lymphatic steps, we chose a new mode of inoculation to infect mice with *L*. *sigmodontis* L3. The intravenous inoculation revealed itself as very successful as shown by the recovery rate (*i*.*e*. the percentage of recovered worms compared to the number of inoculated L3). Twice as many larvae were recovered in the pleural cavity of IV-inoculated mice compared to naturally or SC-infected mice. However even in IV-infected mice more than half of the inoculated L3 are lost post-inoculation supporting the hypothesis that a significant portion of the L3 is located elsewhere and do not develop further into gravid adults. In this IV model, all larvae are delivered at the same time in blood, which could explain why so many larvae are recovered in the lung as early as hours after their injection. Indeed, the removal of skin and lymphatic bottlenecks favours a quick synchronized arrival of L3 in the lung. This model also suggests that that migration through the lung is fast as larvae start to accumulate in the pleural cavity as early as 2 hours after injection.

The major differences observed between SC- and IV-inoculated mice could be due to this very early higher number of L3 in the IV-inoculated mice. Regarding the pathology, there is a strong correlation between the number of haemorrhages and the number of L3 recovered in lungs, the highest number of haemorrhages being observed at h6 in IV-infected mice. Lung capillaries blocked by L3, as well as the movement of the L3 themselves, could cause the haemorrhagic areas observed in the lung. Such a correlation is not true for neither the presence of granulomas nor the accumulation of neutrophils in the perivascular space of lungs. These two phenomena are observed much later in the infection (d4 and d8), when the number of larvae in both IV- or SC- infected mice are similar.

This is accompanied by an increase in pro inflammatory cytokines (Fig 7) such as IL-1β and IL-33 (a crucial cytokine for Th2-mediated host defense playing a central role in controlling immune responses in barrier tissues [50,51]). The second phase, which occurs 4–8 days post infection, is characterized by the presence of granuloma and neutrophils in the perivascular spaces of lungs (Fig 3), an accumulation of L3 in the pleural cavity (Fig 1) and an increase of neutrophils, eosinophils and macrophages in both the bronchoalveolar and pleural spaces (Fig 4). This is associated with an increase of regulatory cytokines such as IL-9 (a pleiotropic cytokine involved *inter alia* in the ability of many cells to regulate inflammation and immunity by affecting many cell types [52]) in lung transcripts or protein levels of IL -4 and MCP-1 in bronchoalveolar lavage. In addition, transcripts of proinflammatory cytokines, such as IL-6, CXCL1 and CXCL5, were downregulated (Fig 7). Consistent with this expression pattern, treatment with anti-CXCL5 antibody attenuates lung neutrophil accumulation in rodent models of lung inflammation [53].

In *L*. *sigmodontis*-infected mice, neutrophilic infiltrates were observed in the lung capillaries as early as h6 p.i. and were resolved by d4. At that time point and up to d8 neutrophils are seen in the lung perivascular spaces. Neutrophils can promote the development of alternatively activated macrophages [54]. Then the macrophage immunoregulatory phenotypes that develop during filarial infection can divert the early immune response to induce the repair of injuries to the lung tissue caused by infective larvae. Following the arrival of L3 in the pleural cavity, recruitment of neutrophils in the pleural space through CXCL1 chemoattraction, working in coordination with other cell populations, including eosinophils and macrophages, could target the infective larvae. These cells contribute to the genesis of pleural granuloma, a cellular reaction mounted against the worms that would gradually eliminate them [5].

A large part of these intrapulmonary neutrophils also showed high level of intracellular S100A9, and both S100A8 and S100A9 transcripts are detected within hours in the lung of infected mice. No monocyte/macrophage or any other mononuclear cells were observed expressing S100A9 in the lung of *L*. *sigmodontis* infected mice. Intracellular proteins S100A8 and S100A9 belong to the large group of S100 calcium-binding proteins and form a heterodimer, calprotectin, (also called Mrp8/14-complex or S100A8/A9) which is abundant in neutrophils. This complex represents 40 to 60% of the neutrophil cytosolic granules content and has been shown to be present in lung pathologies [55–57] as well as in onchocercian nodules in which neutrophils are recruited [58]. Moreover, S100A8 and S100A9 are essential in the response to vascular injury [59] and this process is likely to happen in the lung of *L*. *sigmodontis* infected mice in which L3 exit the capillaries, thus damaging the endothelium. The release mechanism and the mode of action of calprotectin by neutrophils remains unknown in *L*. *sigmodontis* infected mice. However, S100A8 and S100A9 are known to be released from neutrophils as part of Neutrophil Extracellular Traps (NETs), during NETosis, promoting the anti-infectious activity of neutrophils [60]. Neutrophils can sense microbe size and selectively release NETs in response to large pathogens such as fungi [61]. The NETs have been shown to trap the larval nematode *Strongyloides stercoralis* facilitating parasite killing by cells of the immune system [62], but their role on filarial L3 has not been fully evaluated yet, although two recent studies underline the induction of NETS by filariae. We have shown that *L*. *sigmodontis* L3 are able to promote the release of neutrophil extracellular traps *in vitro* [63]. Also, a mechanism of NETosis has been demonstrated in human onchocerciasis with an induction via *Wolbachia* endobacteria and direct ligation of *Wolbachia* lipoprotein by neutrophil TLR2/6 [64]. Thus, the release of S100A9 could be due to two phenomena: i) one in which S100A9 is a danger signal released in response to the presence of a filarial pathogen and is responsible for cell recruitment, and ii) one which is linked to NETosis and to the release of the content of the cytosolic granules. These two circumstances could be additive and the NETosis could lead to the release of more S100A9, both mechanisms contributing to both parasite killing and tissue repair.

Other parasitic Nematodes have been reported to have similar patterns of lung migration, in particular *Nippostrongylus brasiliensis*. Although *N*. *brasiliensis* is a Clade V gastrointestinal parasite of rats, its L3 penetrate through unbroken skin and migrate to the lung where the third moult occurs. Similar lung damage has been reported in mice infected with *N*. *brasiliensis* by d2 p.i., which were resolved by d7 [54]. In addition, extensive neutrophil inflammation was observed in this nematode infection [54]. Furthermore, many inflammatory cytokines were detected: IL-1β, IL-17, potentially involved in the increase of the number of neutrophils, but also IL-4 and IL-5. These interleukins could alternatively activate the alveolar macrophages and block the acute lung injuries by limiting IL-17 production by RELM-α, YM-1 and arginase [65]. It is interesting to point out that even if these two very different species of nematodes (a tissue-dweling filariae versus a gastrointestinal strongyle roundworm) present a common behaviour regarding the migration of L3, there are many important differences. One of the major distinctions between the species is the damage to the lung during an infection. Whereas infection by *N*. *brasiliensis* can result in the development of COPD and emphysema, the presence of *L*. *sigmodontis* in the lung is rather asymptomatic, which could also explain the lack of documentation on this early phase. Much of the pathology associated with filariasis has indeed been correlated with the presence of microfilariae during the patent phase. Mature gravid filariae periodically release microfilariae which can be trapped within the pulmonary microcirculation [44]. The degenerating microfilariae then release their antigenic constituents which triggers a specific immune response known as tropical pulmonary eosinophilia [66].

Even though migrating filarial L3 cause only transient damage to the lung, further investigation would be helpful to decipher the consequences of such injuries on the development of chronic filariasis including the behaviour of released microfilariae in this pleural environment.

## Supporting information

S1 Fig L3 tissue repartition. Overview from [2,11,12,42,43] and current data (from Fig 1). L3 were recovered from either mice, jirds or cotton rats; number of recovered L3 were normalized as F/L3 and pooled per time point. SC: subcutaneous tissue; Lymph: lymph nodes; Pleural: pleural cavity.
(TIF)
