# Supplementary figures and images for: Migratory phase of Litomosoides sigmodontis filarial infective larvae is associated with pathology and transient increase of S100A9 expressing neutrophils in the lung
Source: PLoS Negl Trop Dis. 2017 May 9;11(5):e0005596. doi: 10.1371/journal.pntd.0005596 (PMC5438187; doi:10.1371/journal.pntd.0005596)

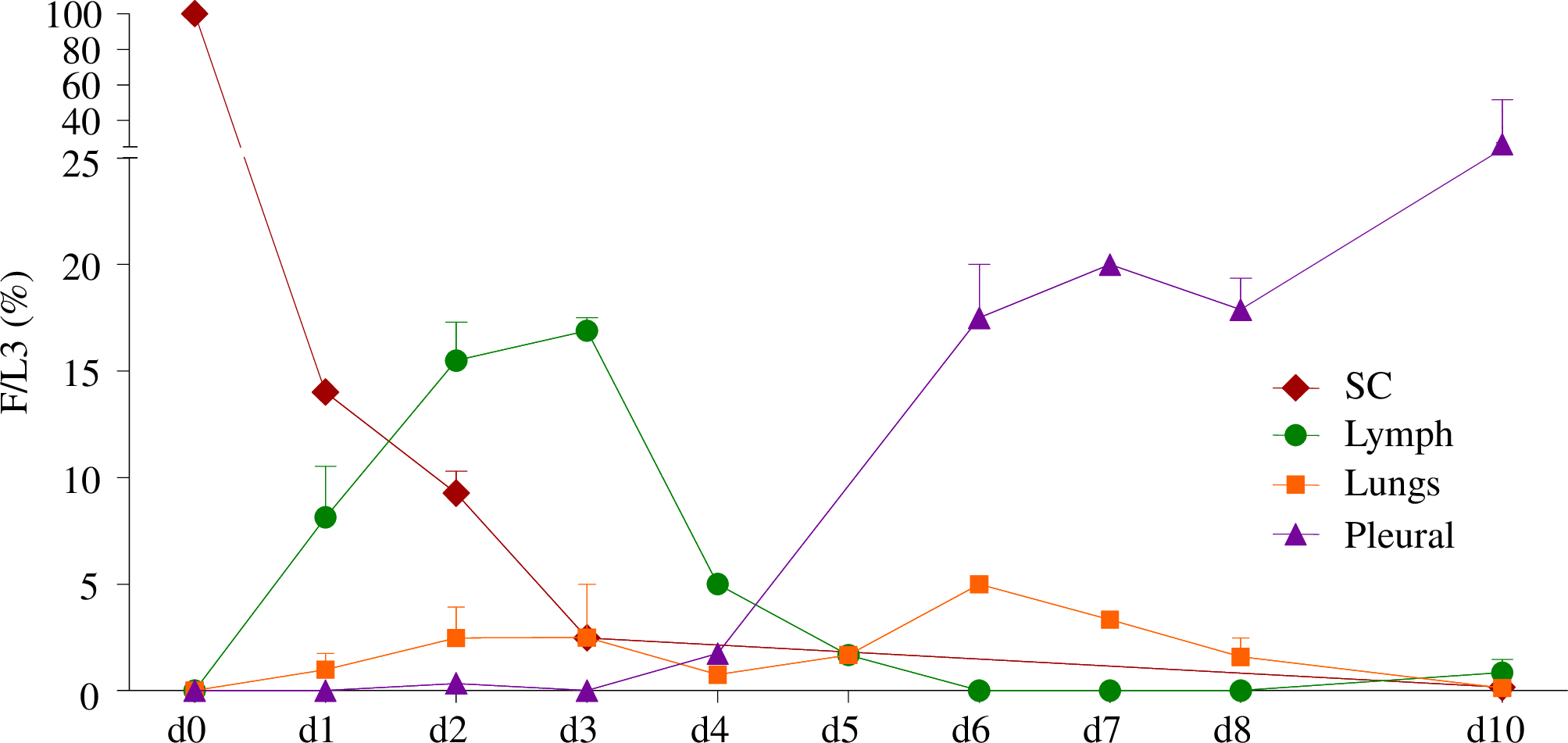

Supplement: S1 Fig — Overview from [2,11,12,42,43] and current data (from Fig 1). L3 were recovered from either mice, jirds or cotton rats; number of recovered L3 were normalized as F/L3 and pooled per time point. SC: subcutaneous tissue; Lymph: lymph nodes; Pleural: pleural cavity. (TIF) [file pntd.0005596.s001.tif]
